# Parameter-free representations outperform single-cell foundation models on downstream benchmarks

**Published:** 2026-02-18

**Authors:** Huan Souza, Pankaj Mehta

**Affiliations:** 1 Department of Physics, Boston University, Boston, MA, 02215, USA; 2 Faculty of Computing and Data Science, Boston University, Boston, MA, 02215, USA

## Abstract

Single-cell RNA sequencing (scRNA-seq) data exhibit strong and reproducible statistical structure. This has motivated the development of large-scale foundation models, such as TranscriptFormer, that use transformer-based architectures to learn a generative model for gene expression by embedding genes into a latent vector space. These embeddings have been used to obtain state-of-the-art (SOTA) performance on downstream tasks such as cell-type classification, disease-state prediction, and cross-species learning. Here, we ask whether similar performance can be achieved without utilizing computationally intensive deep learning-based representations. Using simple, interpretable pipelines that rely on careful normalization and linear methods, we obtain SOTA or near SOTA performance across multiple benchmarks commonly used to evaluate single-cell foundation models, including outperforming foundation models on out-of-distribution tasks involving novel cell types and organisms absent from the training data. Our findings highlight the need for rigorous benchmarking and suggest that the biology of cell identity can be captured by simple linear representations of single cell gene expression data.

## I. INTRODUCTION

Advances in single-cell transcriptomics have transformed our ability to discover and experimentally characterize cell types across tissues and organisms [1–15]. A major achievement of this research program has been the creation of “cell atlases” that catalogue cellular gene expression with single cell resolution [9, 16]. Cell atlases often contain data from hundreds of million cells, with the amount of data expected to grow as sequencing costs continue to drop and new experimental techniques and modalities emerge [17]. For this reason, there is immense interest in leveraging single cell RNA-sequencing (scRNA-seq) data to extract biological insights about the molecular basis of cellular identity.

Although every cell in an organism is genomically identical, different cell types express different subsets of genes, giving rise to observed phenotypic and functional differences. One seductive idea is that the essential aspects of cellular identity are encoded in the statistical properties of cellular gene expression profiles. If true, this suggests that learning good representations of single-cell data offers a powerful route for understanding cellular identity across cell types, tissues, and species. This perspective has motivated a broad range of statistical approaches for analyzing scRNA-seq datasets.

The hope that complex statistical models can be used to learn new biology is buttressed by the tremendous success of protein language models at tasks such as sequence-structure prediction (see [18, 19] for recent reviews). Motivated in part by this work, several works have trained large self-supervised “foundation models” on cell atlas data [20–27]. Models such as TranscriptFormer use transformer-based architectures to learn a generative model for gene expression (i.e. mRNA counts) by embedding genes into a latent vector space. Gene embeddings from foundation models have been used to obtain state-of-the-art (SOTA) performance on downstream tasks such as cell-type classification, disease-state prediction, and cross-species learning. This is often cited as evidence that models like TranscriptFormer learn general-purpose biological representations of gene expression that can serve as inputs for more ambitious “virtual cell” models [28].

Still, it remains unclear if the representations learned by foundation models capture biological structure beyond that which is already present in appropriately processed scRNA-seq data [29, 30]. A growing body of work has shown that simple, interpretable methods can perform remarkably well across diverse single-cell analysis tasks. For example, linear and physics-inspired approaches such as Single-cell Type Order Parameters (scTOP) enable accurate cell-type classification, interpretable visualization of developmental dynamics, and principled analyses of cell fate transitions without requiring large-scale model training [31–36]. These observations raise two basic but under-explored questions: how complex is the structure of scRNA-seq data itself? What level of representational sophistication is required to extract the biologically relevant variation captured by current benchmarks?

There are several good reasons to believe that embeddings from single cell foundation models may be less powerful than those from protein language models [37, 38]. In contrast to protein sequences, which are discrete, high-quality, and tightly constrained by biophysics, scRNA-seq data are sparse, noisy, and characterized by substantial technical variability, including dropout effects and batch-specific artifacts [39]. Moreover, cellular identity reflects a complex interplay between gene expression, signaling, environmental context, and post-transcriptional regulation, rather than being fully specified by transcript counts alone. This is in stark contrast with proteins where the information needed to determine its three-dimensional structure is contained almost entirely in its sequence (Anfinsen’s principle) [40–43].

**FIG. 1:**
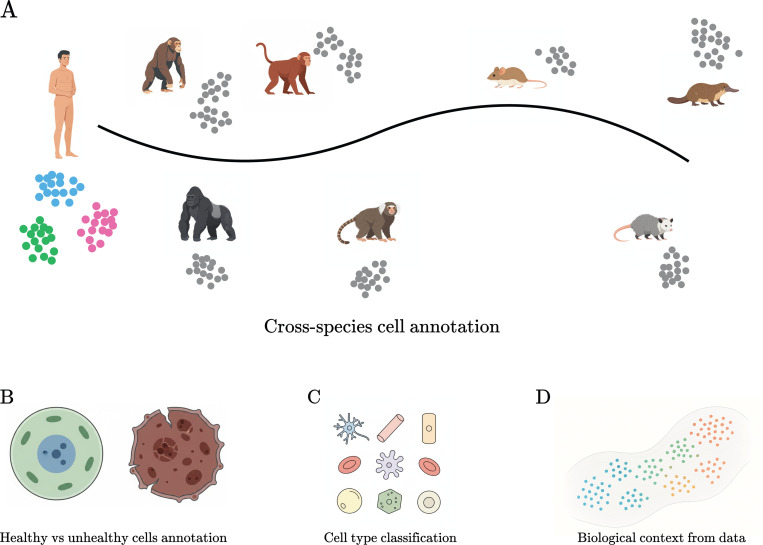
Downstream Tasks/Benchmarks analyzed in this paper. **A.** Cross species cell annotation. The goal of this task is to use labeled cells from one species (e.g. humans) to annotate cell types in another species. **B.** Discrimination between healthy and infected cells. **C.** Cell type classification. **D.** Extracting biological context (i.e. gene-TF interactions) from data.

Inspired by these considerations, we systematically compared the performance of simple, interpretable pipelines to the reported performance of large scale single-cell foundation models on common downstream tasks (see Fig. 1) [29]. We find that by carefully choosing pre-processing steps and normalization procedures, it is possible to achieve SOTA or near-SOTA performance using simple representations where cells are viewed as vectors in gene expression space. The performance of these pipelines often exceeds that of foundation models, despite the fact that they require orders-of-magnitude less computational resources and have almost no free parameters (See SI.B3 for an extended comparison).

These results motivate a unifying interpretation: much of the biologically relevant structure present in current scRNA-seq benchmarks is already accessible through low-complexity linear representations. Consequently, widely used evaluation tasks primarily reflect intrinsic properties of the data manifold^1^ rather than the discovery of new biologically meaningful structure. Our work highlights the need for more stringent evaluation protocols and provides strong evidence that simple representations in terms of normalized gene expression counts capture many of the key aspects of cell identity contained in scRNA-seq cell atlases.

## II. KNOWLEDGE TRANSFER ACROSS SPECIES

Cross-species cell-type annotation provides a natural test of how biological identity is encoded in single-cell transcriptomic data. Because species differ in gene content, regulatory architecture, and evolutionary history, successful transfer requires isolating gene-expression programs that are conserved across evolution and robust to organism-specific variation. For this reason, it is commonly assumed that successfully completing this task, especially on novel species and cell types absent from the training data, requires complex statistical models.

For example, the authors of [27] used a “ transfer learning framework in which labels from one species (source species) [were] transferred and evaluated on embeddings from a different species (target species)” to analyze a spermatogenesis dataset consisting of seven cell types (Sertoli cells, early spermatid, late spermatid, male germ line stem cell, somatic cell, spermatocyte, spermatogonium) and eight mammalian species (Human, Chimpanzee, Rhesus, Marmoset, Gorilla, Mouse, Platypus, Opossum). They interpreted the success of TranscriptFormer at this task as evidence that their embeddings successfully capture hard-to-learn evolutionary relationships between genes and cell types [44].

**FIG. 2:**
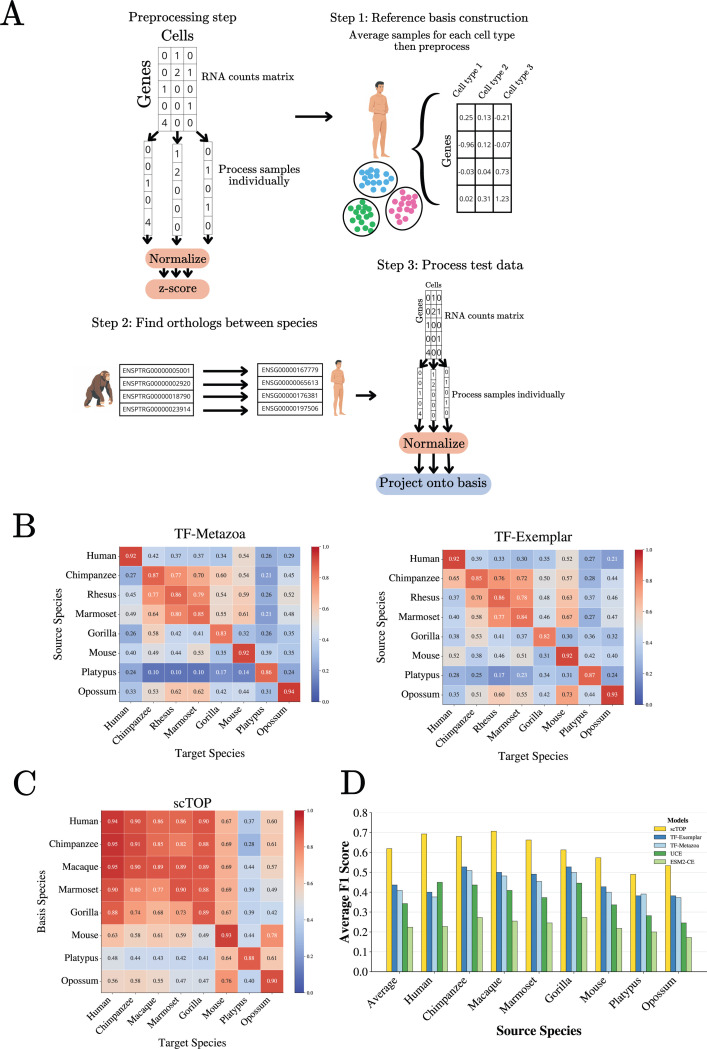
Cross-species transfer learning on novel organisms and cell types. **A.** Pipeline used by *parameter free*, linear algebra-based method scTOP [31] to perform cross-species annotation on the spermatogenesis dataset [27, 44]. **B.** Transfer matrix of macro F1 scores for testis cell type classification across mammals for the TranscriptFormer foundation models TF-Exemplar and TF-Metazoa as reported in [27]. **C.** Transfer matrix of macro F1 scores for testis cell type classification using scTOP. **D.** Comparison between scTOP and foundation models on the cross-species annotation task (F1 scores for foundation models are reported in [27]).

The results of this procedure for two TranscriptFormer model architectures, TF-exemplar and TF-Metazoa as reported in [27], are shown in Figure 2b, with larger scores (red) indicating better transfer learning [27]. Notice that in general transfer learning works poorly between humans and other organisms, with F1-scores consistently below 0.5. On the other hand, the models are much more adept at transferring knowledge across chimpanzee, rhesus, and marmoset. This is true despite the fact that chimpanzees are evolutionarily closer to humans (approx 4–6 million years) than marmosets (35–40 million years) [45].

To better understand the difficulty of this task, we re-analyzed this dataset using scTOP [31]. scTOP is a simple linear-algebra based method with *no free parameters* that constructs a reference basis for cell types of interests and classifies new cells by projecting their gene expression profiles onto this basis. The basic steps involved in scTOP are shown in Fig. 2A. A more detailed description can be found in the Methods, Ref. [31], and the accompanying Python notebooks at our Github repository. Briefly,

- The gene expression of each cell is *normalized to itself*, by converting mRNA counts to z-scores that reflect the rank ordering of genes within the cell (e.g. a gene whose expression percentile is 50th percentile is assigned a score *z* =0, a gene that is at the 84th percentile a *z* =1, etc). We have found that this normalizing procedure significantly eliminates batch effects because all cells are normalized independently [31].
- A reference basis is created for cell types of interest (source). Due to the extremely noisynature of scRNA seq data, this is done by creating normalized pseudo-bulk expression profiles by averaging the expression profiles of cells with the same source label.
- We assume that the number of genes is greater than the number of source cell types and hence the source cell types define a linear subspace of the full gene expression space.
- To classify a target cell, we calculate the (non-orthogonal) linear projection of the target cellon each of the source basis vectors. The target cell is labeled as the source cell type with the largest projection.

In order to compare cell types across species, we restricted each dataset to orthologous genes and mapped all orthologs to their basis species counterparts for consistency (Fig. 2). This allows us to define a common coordinate system in gene space across species. This step is inherently lossy. While the human dataset retains 34,168 genes, other organisms are reduced to roughly 14,000 shared orthologs. We also restrict ourselves to eight mammalian species in the original spermatogenesis dataset [44] that could be directly accessed from the TranscriptFormer Github tutorial using their built-in data processing function [46] (see Methods).

Across all eight species, scTOP achieves consistently higher macro F1-scores than foundation models (Fig. 2C). Importantly, this improvement persists even for evolutionarily distant species pairs, including transfers involving platypus. These findings indicate that the conserved biological structure relevant for cross-species annotation is already strongly accessible through linear representations once appropriate normalization and orthology mappings are applied. These results suggest that the gene expression profile characterizing a cell type is extremely conserved. It further implies that the “data manifold” spanned by biologically-realized cell types in gene expression space can be well-approximated as a linear subspace. A practical consequence of these observations is that one can reliably use a simple pipeline to annotate cells of one species using cells from another species.

## III. IDENTIFYING GLOBAL BIOLOGICAL STRUCTURE FROM DATA

We also wanted to understand if simple vector representations of gene expression can capture global biological relationships between cell types and species. In [27], it was argued that one way of assessing this is by looking at the cosine similarity between embeddings for different cell types and species. In a good model, the cosine similarity between cell types at similar developmental stages should be larger than the similarity between cell types at more distant stages. The similarity between species-level embeddings should also decay with evolutionary distance.

**FIG. 3:**
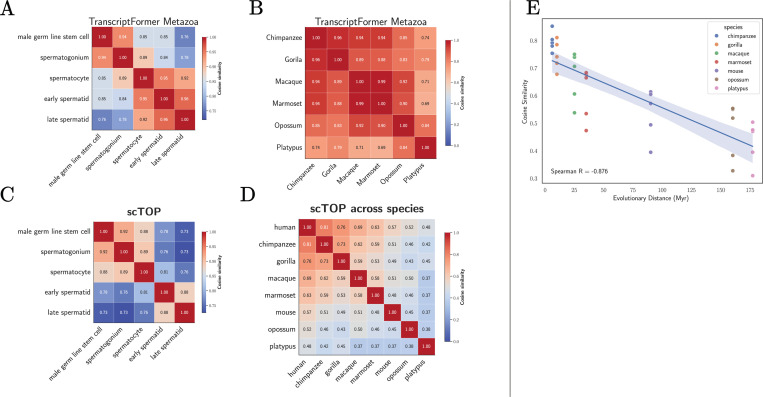
Biological context from data. Cosine similarities for embeddings from TF-Metazoa and scTOP for different male germline developmental lineages and species. **A.** TranscriptFormer germline; **B.** TranscriptFormer species; **C.** scTOP germline; and **D.** scTOP species. **E.** Cosine similarity from scTOP between humans and indicated species as a function of evolutionary distance.

This analysis was carried out using embeddings from TranscriptFormer for the spermatogenesis dataset in [27] and the results are shown in Fig. 3A. As expected, the cell types at earlier developmental stages before meiosis is complete (male germ line stem cell, Spermatogonium, Spermatocyte) are more similar to each other than cells at later developmental stages where cells are haploids (early spermatid, late spermatid). Similarity scores between species-level embeddings from TranscriptFormer also seem to decay with evolutionary distance, though this signal is much less pronounced (Fig. 3B).

Using the same dataset and evaluation protocol as [27], we analyzed representations generated by the scTOP preprocessing pipeline (Fig. 2A). For each cell type, we constructed an average gene expression vectors using pseudo-bulk gene counts as described in the last section and computed cosine similarities (Fig. 3C). As expected, within the male germline developmental lineage, we observe that cell types from successive developmental stages are more similar to each than cells types from more distant stages. Comparing to TranscriptFormer embeddings, it is visually clear that early and late developmental stages are more distinct in linear representations than the in embeddings from foundation models.

To compute the cosine similarity between different species, we averaged the gene expression profiles of cells from each species to compute a single representative species-level gene expression vector. In our pipeline, we restricted our analysis to gene orthologs and cell types shared by all species. As can be seen in Fig. 3D, our species-level gene expression vectors exhibit a much stronger evolutionary signal than embeddings from TranscriptFormer, with evolutionarily related species having much higher similarity than distant ones. The ability of appropriately normalized gene expression vectors to capture evolutionary relationships is also evident in Fig. 3E, which shows the cosine similarity of different species as a function of evolutionary distance from humans. We find that cosine similarity and evolutionary distance are highly anti-correlated, with a very strong Spearman-correlation of *R* =−0.876.

Collectively, our findings show that conserved developmental and evolutionary relationships between cell types and species are already quantitatively captured by properly normalized gene expression vectors and do not require the use of any statistical models or fitting.

## IV. TABULA SAPIENS CELL TYPE CLASSIFICATION TASK

Human cell-type annotation provides a complementary setting to cross-species transfer for probing the nature of scRNA-seq data. Unlike evolutionary transfer, this task focuses on discriminating a large number of closely related cell types within the same organism, often in the presence of substantial technical noise and dropout. In [27], TranscriptFormer and other single-cell foundation models were benchmarked on this task using the Tabula Sapiens 2.0 dataset [47]. This was done by training a tissue-level logistic regression classifier for cell identity on embeddings from each of the foundation models and then assessing the classifier on an independent test dataset of cells from the same tissue.

We asked whether similar performance could be achieved with simpler pre-processing pipelines. This prediction task involves distinguishing many more cell types than the cross-species annotation task in the last section. For example, the immune tissue in Tabula Sapiens 2.0 dataset has more than 50 distinct cell types, many of which are closely related. As the number of cell types increases, the difficulty of the classification task increases dramatically, especially when the underlying data is noisy. This takes on added significance for the Tabula Sapiens 2.0 dataset, which in our experience, has much higher levels of technical noise and dropout than the spermatogenesis dataset analyzed above (see Supporting Information for a discussion of batch effects in heart cell types as a typical example B5).

In this noisier setting, raw linear projections alone are insufficient (see SI B2). For this reason, we extended our pipeline by introducing two additional denoising steps, ANOVA-based gene selection and Principle Component Analysis (PCA), and replacing linear projections by a logistic regression-based classifier analogous to the one used to benchmark the single cell foundation models (see Methods for detailed explanation). These operations aim to isolate the most informative directions of variation while retaining simplicity and intepretability.

To perform our analysis, following [27] we selecte all cell types from each tissue that have at least 100 cells and separate the data into training and test sets (80–20 ratio). We normalize the gene expressions of each cell independently as described above. We then perform an ANOVA on each tissue to select the 20000 genes that vary the most across cell types, followed by another normalization step to standardize the gene expression profile of each cell. We then further denoise the data by performing a PCA and projecting the data onto the 220 most variable PCA components (see methods section A9 for a discussion on the choice of hyper-parameters). The ANOVA and PCA results from the training data sets are also applied to the test set to avoid information leakage. These de-noised cellular gene expression profiles play the same role in our pipelines as the cellular embeddings derived from the single-cell foundation models and serve as inputs to a logistic regression classifier (see Methods).

**FIG. 4:**
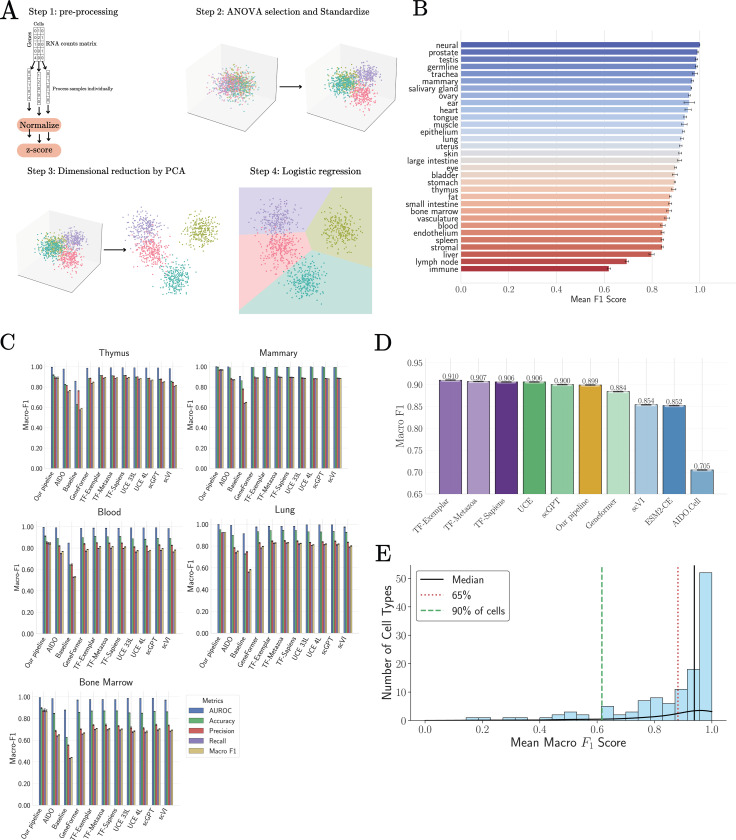
Tabula Sapiens 2.0 cell type classification task. **A:** Overview of our pipeline. **B:** Per-tissue results for Tabula Sapiens 2.0 classification task for pipeline. **C:** Detailed results and comparisons between our pipeline and foundational models as reported in [29]. **D:** Average result per-tissue-pertype cell classification for Tabula Sapiens 2.0. **E:** F1-scores distribution per cell type for our pipeline.

Classification performance is evaluated using five-fold cross-validation. Figure 4B shows the per-tissue macro F1-scores obtained using this pipeline. Across 24 of 31 tissues, macro F1-scores exceed 0.8, indicating that a large fraction of the discriminative structure is captured by this representation. Tissues with low scores, such as blood and immune, generally have many cell types that are closely related (e.g. different variant of T cells) and are also difficult to classify using single cell foundation models. For direct comparison with foundation models, we focus on the five tissues for which benchmark results are publicly available through the CZI benchmark portal [29]. As shown in Fig. 4C and Fig. 4D, performance obtained with our pipeline closely matches (and in some cases exceeds) that reported for large foundation models, including TranscriptFormer. The mean macro F1-score across tissues is 0.899, compared to 0.910 and 0.907 for TranscriptFormer variants. Notably, the remaining performance gap is largely driven by a small subset of particularly difficult cell types with strong transcriptional similarity (see B1).

Figure 4E shows the distribution of macro F1-scores across individual cell types. More than half of all cell types achieve scores above 0.9, while the overall mean is dominated by a minority of hard-to-classify populations. This pattern suggests that, for most cell types, the relevant biological distinctions are already linearly accessible once noise is appropriately suppressed.

Together, these results indicate that human cell-type annotation benchmarks primarily probe the effectiveness of denoising and feature selection, rather than the discovery of complex non-linear representations. Within the regime explored by current datasets, pipelines that isolate informative variance directions capture most of the structure required for accurate classification.

## V. IDENTIFYING CELLS INFECTED BY SARS-COV-2

**FIG. 5:**
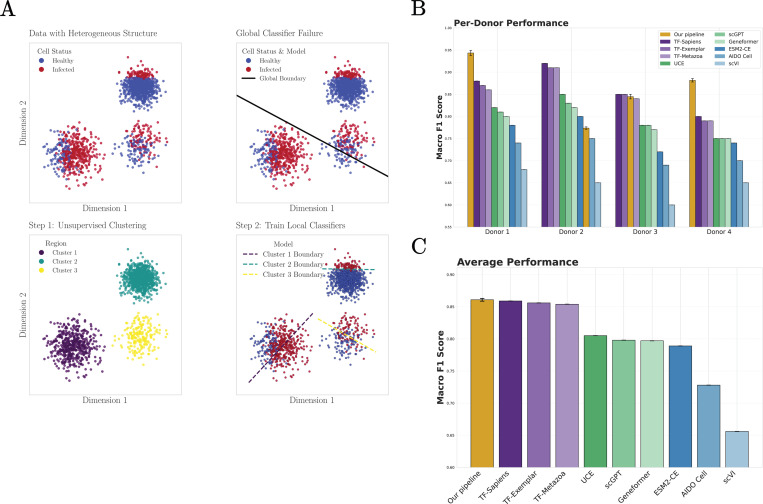
Identifying cells infected by SARS-CoV-2. **A.** Schematic illustrating why local classifiers are necessary for this task. **B.** Comparison between our pipeline (yellow) and foundation models (as reported in [27]) at classifying SARS-CoV-2 infected and uninfected cell from four distinct donors (data from [48]). **C.** Comparison of average disease state prediction F1 scores of uninfected and infected cells across all tissues and donors for our pipeline and foundation models.

We next consider the task of classifying SARS-CoV-2–infected versus uninfected cells across multiple immune and lung cell types, using the dataset introduced in [48] and previously analyzed in [27]. Distinguishing disease states within scRNA-seq data is a subtle task that presents new difficulties. Healthy and infected cells of the same cell type are highly correlated. There are also strong cell-type–specific class imbalances, with some populations containing nearly equal numbers of infected and uninfected cells and others being overwhelmingly composed of healthy cells. These two challenges make it difficult to learn a single, global classifier that can distinguish infected and uninfected cells across all cell types (see Fig. 5a).

To address these challenges, we augment the pipeline used in the last section to annotate human cell types (Fig. 4a) with an additional unsupervised clustering step (Fig. 5a). After normalizing the data, performing ANOVA gene selection and standardization, we used PCA to perform dimensionality reduction and apply the Leiden clustering algorithm to partition the low-dimensional space into roughly 15 biologically coherent regions (clusters). Critically, this clustering operates without knowledge of health status and discovers natural subdivisions in the data based purely on transcriptional similarity.

We then trained a separate logistic regression classifier within each cluster. This allows our pipeline to learn local classifiers that identify different context-dependent signatures of infection (see Fig. 5A). The key insight behind this strategy is that disease states are not globally separable, but locally distinguishable. Within a cluster of related cells, for example alveolar macrophages from similar tissue environments, the differences between healthy and infected states become pronounced. A classifier trained on this local structure can detect these differences without being confounded by the larger-scale variation across cell types and tissues.

Our results demonstrate the power of this approach. For the SARS-CoV-2 dataset [48], across all donors our method achieves a macro F1-score of 0.862, exceeding the performance of foundation models (Fig.5 B,C). These results further support a consistent interpretation across tasks: when biologically relevant signals are locally structured, extensions of linear pipelines that respect this locality are sufficient to recover disease-associated variation. Rather than requiring highly expressive global embeddings, accurate disease-state identification in this setting depends primarily on isolating the appropriate scale at which biological differences are expressed.

## VI. STATISTICAL STRUCTURE OF GENE-GENE AND TF-TARGET RELATIONSHIPS

Single-cell foundation models such as TranscriptFormer have been used to identify interactions between transcription factors (TFs) and protein-coding genes. The approach works by using the model to generate synthetic gene expression profiles, then calculating how often a TF and target gene appear together in these generated profiles [27]. This has been touted as a key advantage of foundation models: they can serve as a “virtual instrument” for probing gene regulatory relationships that would be difficult to study experimentally.

However, it remains unclear whether this capability is unique to foundation models or whether similar insights can be obtained directly from the data itself. To test this, we repeated the analysis from [27], but instead of using cellular embedding vectors from TranscriptFormer, we used the normalized gene expression profiles from scTOP as inputs. Briefly, for each transcription factor, cells with high TF activity are identified, and the probability of observing each gene in these cells is compared to its marginal expression probability across the whole dataset. This defines a Pointwise Mutual Information (PMI)-inspired enrichment score that quantifies conditional dependence between TF activity and gene expression. Statistical significance is assessed using standard Z-scoring and false discovery rate correction (see Methods).

**FIG. 6:**
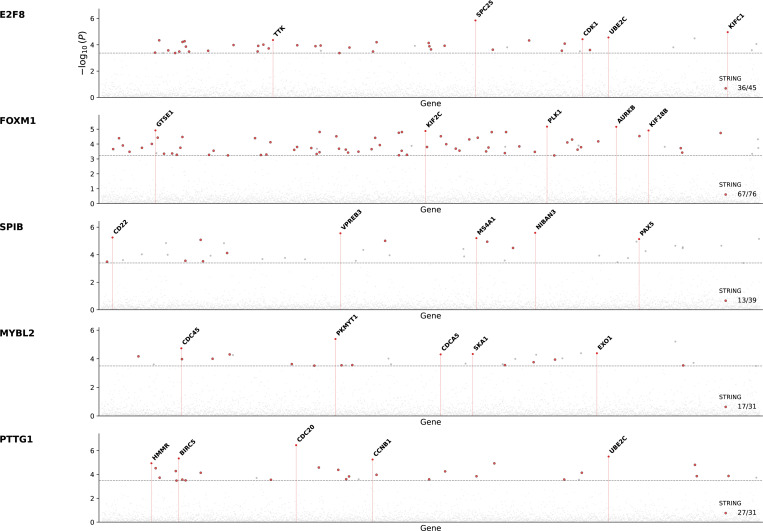
Validation of inferred TF-gene associations. Each point represents the Pointwise Mutual Information (PMI)-enrichment score between the indicated transcription factor (row name) and a protein coding gene. PMI scores are calculated using normalized scRNA-seq gene expression profiles from scTOP. Points lying above that dashed line indicate predicted interactions that are statistically significant after FDR correction, with points colored red indicating a protein-protein interactions that has been validated using the STRING database [49].

We find that these empirically computed conditional statistics recover coherent and biologically meaningful TF–gene association patterns. When validated against independent protein–protein interaction evidence from the STRING database [49], significant associations are enriched well above background expectations (Fig. 6). This indicates that regulatory structure is already encoded in the observable conditional co-expression patterns of normalized scRNA-seq data, without requiring higher-order modeling or latent representations.

## VII. BENCHMARK SATURATION AND NEAR-LINEAR TRANSCRIPTIONAL GEOMETRY

One of the surprising results of our analysis is the empirical observation that across diverse tasks – cross-species transfer, within-species cell-type classification, disease-state prediction, and biological context extraction – simple linear representations of cells in terms of normalized gene expression profiles can achieve SOTA or near SOTA performance comparable to extremely expressive single-cell foundation models. Here we provide a potential explanation for this observation by thinking about gene expression datasets from a geometric viewpoint.

We view each cell as a point in gene space, with each gene is a different coordinate dimension (analogous to three-dimensional physical space). The position of a cell along each axis is just the normalized expression of that gene in the cell. For scRNA-seq data where one simultaneously measures tens of thousands of genes, this space is extremely high-dimensional. However, a key insight is that the effective space spanned by biologically realized cells is much lower-dimensional. The reason for this is that the set of biologically realized gene expression profiles across cells and species is a very small subset of all possible gene expression profiles.

In principle, this low-dimensional space could be highly structured. If this were the case, then representing cells would require extremely expressive models that can learn this complex structure. However, the empirical observation that simple linear representations work well on downstream tasks led us to hypothesize that the submanifold corresponding to real cells is well-approximated by a *linear subspace*. To test this hypothesis, we created low-dimensional embeddings of our datasets using Isomap [50], an algorithm explicitly designed to model low-dimensional subspaces as curved manifolds, and compared them to low-dimensional embeddings obtained from PCA, which explicitly assumes that data lives in a linear subspace.

**FIG. 7:**
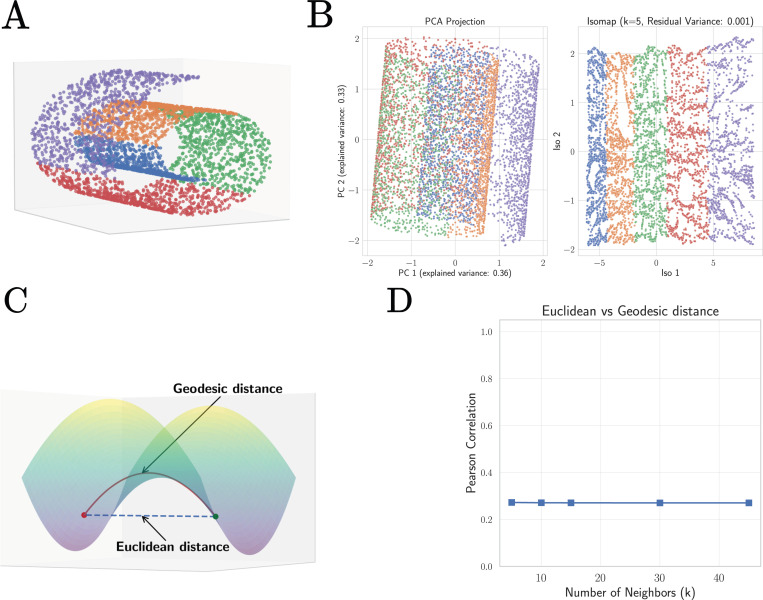
Detecting non-linear structure in data using geodesic distances from Isomap. **A.** Swiss roll dataset in three dimensions. **B.** Two dimensional projections of the swiss roll dataset using Principle Component Analysis (PCA) and the non-linear manifold learning technique Isomap [50] which uses the geodesic distance between points to create embeddings. **C.** Schematic illustrating difference between geodesic and Euclidean distance. **D.** Pearson correlation between Euclidean distances and geodesic distances between data points in the swiss roll dataset as a function of the Isomap hyperparameter *k* (number of nearest-neighbors).

For highly structured but low-dimensional data like the swiss roll dataset shown in Fig. 7A, these two algorithms produce embeddings with extremely different properties. In stark contrast to PCA which simply projects that data to a lower-dimensional representation, Isomap preserves geodesics distances, allowing it to identify the two-dimensional nature of the swiss roll dataset despite its high curvature Fig. 7B. This suggests that one reasonable proxy for assessing if a subspace is curved is to compare geodesic distances between data points obtained from Isomap embeddings with standard Euclidean distances in gene space (see Fig. 7C). As can be seen in Fig. 7D, for datasets like the swiss roll that occupy a highly curved submanifold, Euclidean and geodesic distances have a very low correlation. In contrast, for datasets that can be well approximated as a linear subspace this correlations should be close to one.

**FIG. 8:**
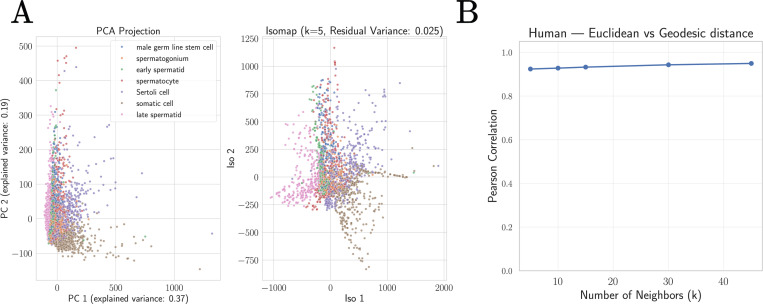
Single-cell scRNA-seq datasets are approximately linear. **A.** Two dimensional embeddings of the human spermatogenesis dataset [27, 44] using Principle Component Analysis (PCA) and Isomap. **B.** Correlation between geodesics and Euclidean distance as a function of the Isomap hyperparameter *k* (number of nearest-neighbors). This correlation is higher than 0.9, providing strong evidence that the subspace spanned by the human spermatogenesis dataset is approximately linear.

To quantitatively assess whether biologically-realized cellular gene expression profiles are well approximated by a linear subspace, we used PCA and Isomap to create low-dimensional embeddings of the human spermatogenesis dataset analyzed above. This choice is inspired by our observation that this data is especially high quality, with relatively little technical noise (see SI B4). As can be seen in Fig. 8A, the resulting low-dimensional embeddings are qualitativly similar. To more directly probe the geometry of this dataset, we compared the Euclidean distance between datapoints in the original gene space to the geodesic distance between points as calculated by Isomap. In contrast with the swiss roll dataset where these distances were very weakly correlated, for the spermatogenesis dataset this correlation is greater than 0.9, providing strong evidence in favor of our hypothesis (for an extended discussion on the other datasets and the Tabula Sapiens tissues datasets, see SI B4).

This geometric perspective helps explain why simple pipelines repeatedly match or exceed the performance of substantially more complex foundation models. When the space spanned by biological datasets is approximately linear, additional model expressivity is not translated into performance gains. It also underscores the need for better understanding the structure of scRNA-seq data to assess if complex foundation models are necessary given current datasets.

## VIII. DISCUSSION

Over the last few years, vast amounts of resources have been dedicated to developing single cell foundation models using cell atlases. Advocates argue that these models have the potential to yield fundamentally new insights into gene regulation and cell identity [20–27]. For example, the authors of [27] claim that TranscriptFormer learns “representations with emergent biological properties that surpass previous approaches” and for this reason represents “a paradigm shift in how we interact with cellular data by functioning as a virtual instrument for biological inquiry” [27]. Foundation models like TranscriptFormer are viewed by their proponents as starting points for the even more ambitious goal of creating AI-based virtual cell models that can serve “as interactive knowledge bases capable of simulating complex cellular phenomena” [27].

Here, we present strong evidence suggesting that extremely simple methods with no or few parameters can achieve comparable and, in many cases, exceed the performance of single cell foundation models on downstream tasks such as cross-species annotation, human cell-type classification, disease-state identification, and biological context extraction. For example, we show that a pipeline combining gene orthology information with scTOP [31], a simple *parameter free* linear-algebra algorithm that represents cells by a single normalized gene expression vector, significantly outperforms single cell foundation models on transfer learning cell types to novel species and cell fates (see Fig. 2). This is especially notable since it has been argued that the performance of single cell foundation models on out-of-distribution tasks “provide[s] compelling evidence that broader evolutionary pretraining enhances biological generalization.” The results presented make a compelling case that this is in fact not the case.

One key advantage of simple representations over foundation models is their high level of interpretability. For example, we have used scTOP to great success to visualize developmental dynamics [31] and assess the fidelity of cells engineered using directed differentiation [32, 33, 36]. These pipelines also naturally interface with promising theoretical ideas about cell fate grounded in dynamical systems and statistical physics [51–55]. We have recently used scTOP to analyze cellular differentiation trajectories and identify signatures of different bifurcation classes directly from data [35].

We emphasize that our present analysis is limited to current scRNA-seq atlases, datasets, and benchmarks. It is plausible that other, more complex, tasks may need the increased expressive power of foundation models. More complicated representations may also be necessary for multimodal datasets that combine multiple types of biological measurements. However, due to the noisy nature of high-throughput single cell experiment, even multimodal data may be amenable to simple representations after appropriate preprocessing. This highlights the need for developing simple methods that can be used to benchmark future multimodal foundation models.

To understand why linear representations perform so well on scRNA-seq data, we examined the organization of single-cell data from a geometric perspective. The results in Sec. VII show that while scRNA-seq data do exhibit some degree of non-linearity, these effects are modest. As a result, performance saturates rapidly once noise is suppressed and the dominant sources of variation are isolated. In this setting, increasing model expressivity yields little additional benefit. Importantly, biological signals such as cell identity, disease signature, and tissue context—often thought to require complex representations—are already recoverable through simple linear transformations. This suggests that current foundation models primarily function as sophisticated denoising procedures rather than tools that uncover fundamentally new biological structure.

This raises questions about why the performance of single cell foundation models fails to live up to the lofty expectations of their proponents, especially given the success of transformer-based protein language models at sequence-structure prediction. A key reason for this is that scRNA-seq data is fundamentally different from the DNA sequencing data used for protein modeling. In lieu of discrete, high quality protein sequences, single-cell transcriptomics produces sparse, noisy measurements of continuous variables rife with technical artifacts and biological variability. In this setting, the inductive biases and high expressivity of neural networks may actually work against them. The biology of cell identity is also significantly more complex than protein biophysics. Whereas it is well accepted that a protein’s sequence contains the information needed to specify its structure (Anfinsen’s principle), cell biology is highly contextual and emerges from the complex interplay between transcription, post-translational modification, signaling, and environmental cues.

From this perspective, the field’s rush toward ever-larger foundation models likely needs reassessment. Our results suggest that current scRNA-seq cell atlases simply do not contain the kind of deep structure that justifies the use of complex deep learning models. The potential cost of this misalignment is considerable because of the incredible computational resources needed to train foundation models. Training TranscriptFormer required processing 100+ million cells on 1000 H100 GPUs, an undertaking accessible only to well-funded institutions with a substantial GPU infrastructure. Even deploying trained models requires considerable computational resources and expertise well beyond that possessed by a typical biology lab. For example, extracting gene and cell embedding using TranscriptFormer requires an A100 GPU.

In addition, the complexity of foundation models means that they are necessarily black boxes with limited interpretability. For this reason, methods like scTOP that employ simple representations of cellular gene expression profiles represent a promising alternative to complex, computationally-intensive foundation models.

## Supplementary Material

Supplement 1

## Data Availability

All datasets used in this work were obtained from publicly available repositories. The data used in cross-species analysis and Tabula Sapiens 2.0 were downloaded from TranscriptFormer package [27]. For COVID-19 disease state identification we used the data available in [48].

## Footnotes

1 In this work, as is common in the machine learning literature, a *manifold* refers to the effective low-dimensional structure formed by scRNA-seq data within the high-dimensional gene-expression space. This usage is informal and geometric, emphasizing distances, curvature, and separability.
